# G6PD Deficiency and Hemoglobinopathies: Molecular Epidemiological Characteristics and Healthy Effects on Malaria Endemic Bioko Island, Equatorial Guinea

**DOI:** 10.1371/journal.pone.0123991

**Published:** 2015-04-27

**Authors:** Min Lin, Li Ye Yang, Dong De Xie, Jiang Tao Chen, Santiago-m Monte Nguba, Carlos Sala Ehapo, Xiao Fen Zhan, Juan Urbano Monsuy Eyi, Rocio Apicante Matesa, Maximo Miko Ondo Obono, Hui Yang, Hui Tian Yang, Ji Dong Cheng

**Affiliations:** 1 Department of Internal Medicine, First Hospital Affiliated to Medical College of Shantou University, Shantou, Guangdong Province, People’s Republic of China; 2 Central Laboratory, Chaozhou Central Hospital Affiliated to Southern Medical University, Chaozhou, Guangdong Province, People’s Republic of China; 3 The Chinese Medical Aid Team to the Republic of Equatorial Guinea, Guangzhou, Guangdong Province, People’s Republic of China; 4 Medical Laboratory, Malabo Regional Hospital, Malabo, Equatorial Guinea; 5 Central Blood Transfusion Service, Malabo Regional Hospital, Malabo, Equatorial Guinea; National University of Singapore, SINGAPORE

## Abstract

**Background:**

Glucose-6-phosphate dehydrogenase (G6PD) deficiency and hemoglobinopathies were the inherited conditions found mostly in African. However, few epidemiological data of these disorders was reported in Equatorial Guinea (EQG). This study aimed to assess the prevalence and healthy effects of G6PD deficiency and hemoglobinopathies among the people on malaria endemic Bioko Island, EQG.

**Materials and Methods:**

Blood samples from 4,144 unrelated subjects were analyzed for G6PD deficieny by fluorescence spot test (FST), high-resolution melting assay and PCR-DNA sequencing. In addition, 1,186 samples were randomly selected from the 4,144 subjects for detection of hemoglobin S (HbS), HbC, and α-thalassemia deletion by complete blood count, PCR-DNA sequencing and reverse dot blot (RDB).

**Results:**

The prevalence of malaria and anemia was 12.6% (522/4,144) and 32.8% (389/1,186), respectively. Overall, 8.7% subjects (359/4,144) were G6PD-deficient by FST, including 9.0% (249/2,758) males and 7.9% (110/1,386) females. Among the 359 G6PD-deficient individuals molecularly studied, the G6PD A^- ^(G202A/A376G) were detected in 356 cases (99.2%), G6PD Betica (T968C/A376G) in 3 cases. Among the 1,186 subjects, 201 cases were HbS heterozygotes, 35 cases were HbC heterozygotes, and 2 cases were HbCS double heterozygotes; 452 cases showed heterozygous α-thalassemia 3.7 kb deletion (-α^3.7^ kb deletion) and 85 homozygous - α^3.7^ kb deletion. The overall allele frequencies were HbS 17.1% (203/1186); HbC, 3.1% (37/1186); and –α^3.7^ kb deletion 52.4% (622/1186), respectively.

**Conclusions:**

High G6PD deficiency in this population indicate that diagnosis and management of G6PD deficiency is necessary on Bioko Island. Obligatory newborn screening, prenatal screening and counseling for these genetic disorders, especially HbS, are needed on the island.

## Introduction

Glucose-6-phosphate dehydrogenase (G6PD) deficiency and hemoglobinopathies are widespread human erythrocyte genetic diseases affecting millions of people [1,2]. The geographical distribution of G6PD deficiency and hemoglobinopathies (mainly sickle cell disease and thalassemia) is closely related to the past and present prevalence of malaria because of a selective advantage against malaria infection [3,4]. Individuals with these disorders show reduced malaria infection, disease severity or parasitic burden [4,5].

G6PD deficiency is prevalent in Africa, the Middle East, the Mediterranean and Southeast Asia [3]. Among the approximately 200 G6PD variant alleles have been described in the world, G6PD A^-^ variant (G202A/A376G) predominates in sub-Saharan Africa where it affects 15 to 20% of the African population [6]. A recent study conducted in The Gambia, found that another variant, G6PD Betica (T968C/A376G), was the most common cause of G6PD deficiency in that part of continent [7], which suggested a regional genotypic difference of G6PD deficiency in Africa. Primaquine (PQ) has been the most commonly used 8-aminoquinoline anti-malarial drug. Over the past 60 years, PQ has been used to treat the liver stages (hypnozoites) of *Plasmodium vivax* and *Plasmodium ovale* malaria to prevent relapses, and as a single-dose or short-course gametocytocidal drug with the goal of reducing transmission of *falciparum malaria*. Despite the therapeutic advantages of PQ, the wider use of the drug is restricted by their toxicity profile. The most important adverse effect of PQ is dose-related hemolysis, which could potentially create significant morbidity and undermine confidence in PQ prescription [8]. Therefore, the diagnosis and management of G6PD deficiency is important for malaria control, which will require the wider use of PQ for both reducing *P*. *falciparum* transmission and achieving the radical cure of *Plasmodium vivax*.

Anemia in children continues to be a major public health challenge in most developing countries, particularly Africa [9]. Although iron deficiency is the most common cause of anemia, many other factors, including malaria, vitamin A deficiency and hemoglobinopathies play important roles in different settings. Sickle cell disease is the largest public health concern [10,11]. An estimated 275,000 people in Africa are born with sick cell disease yearly, approximately 85% of the global affected cases [12]. Hemoglobin S (HbS) is a structural variant of healthy hemoglobin (HbA), which results from a GAG>GTG mutation at codon 20 of β-globin gene. Heterozygote individuals (HbAS) are generally asymptomatic, and homozygote individuals (HbSS) have lifelong acute and chronic complications [13,14]. α^+^-thalassemia is the other common hemoglobinopathy in Africa [2]. Heterozygous α^+^-thalassemia causes slight hematological changes and homozygote carriers show mild microcytic anemia. Although these genetic disorders are a major public health problem and social burden for people in endemic regions, few data are available about the genetic frequencies of these disorders in some sub-Sahara regions.

Equatorial Guinea (EQG) is a country located in central Africa, with an area of 28,068 km^2^. It is divided into a mainland territory, which is bordered by Cameroon to the north and Gabon to the east and south, and five small islands, including Bioko, Corisco, Annobón, Elobey Chico and Elobey Grande. Although innovative malaria control programs in EQG have had a marked impact on malaria infection and mortality, malaria due to *Plasmodium falciparum* is still the major public health problem in the country. The World Health Organization (WHO) estimates that the probability of dying among children under 5 years of age in EQG is 205 of 1,000 live births, with 24% of death attributable to malaria [15]. Because of limited local medical resources, no systematic investigation of G6PD deficiency and hemoglobinopathies was reported in the country.

Here, we performed a large-scale molecular epidemiological survey of G6PD deficiency and hemoglobinopathies in 4,144 subjects, which firstly allows a sight into the prevalence and molecular characterization of these genetic diseases on Bioko Island. It is a prerequisite for defining a specific policy for these genetic diseases screening, genetic counseling, prenatal diagnosis and implementation of PQ for malaria on the island.

## Materials and Methods

### Study Area

**Fig 1:**
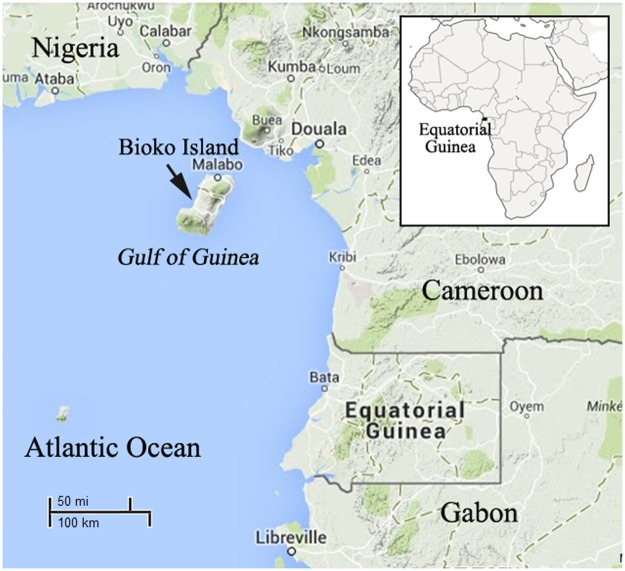
Geographic location of the Bioko Island, Equatorial Guinea.

Bioko Island belongs to EQG and is located in the Gulf of Guinea, about 100 km off the coast of southern Nigeria and 160 km northwest of continental EQG (Fig 1). The island has a population of 266,000 inhabitants (2001 census), including 58% Bubi nationality, 16% Fang nationality, 12% Fernandino nationality and 7% Igbo nationality, they live in the north part of the island. The island has a humid tropical environment. The mean annual rainfall is ~2,000 mm/year. The rainy season starts in May and ends in October with peaks in August and September of ~300 mm/month. Mean daily maximum and minimum temperatures range between 29–32°C and 19–22°C, respectively [16]. The launch of the Bioko Island Malaria Control Project (BIMCP) have had a marked impact on malaria transmission, malaria due to *Plasmodium falciparum* is still the major public health problem on the island. The entomological inoculation rates (EIR) on Bioko Island ranged from 163 to 840, with the outdoor EIRs reaching > 900 infective mosquito bites per year [16], and a malaria prevalence of 52% in 1–4 year old children (2011) [17].

### Population samples

The study population included 4,144 unrelated subjects living on Bioko Island These subjects aged from 1 to 75 years and received the screening for G6PD deficiency and hemoglobinopathies in Malabo Regional Hospital during April 2012 and May 2014. Questionnaires about nationality, gender, age, native status or not and written consent forms were available in Spanish (the most-common language) to ensure comprehensive understanding of the study objectives. This study was approved by the ethics committees of Malabo Regional Hospital and the First Affiliated Hospital of Shantou University Medical College. All subjects or their parents gave their informed consent by signature or thumbprint.

Blood samples were collected with EDTA-K_2_ anti-coagulated tubes from each subject according to the standard procedures described previously [18], and 200uL of blood was adsorbed on Whatman 903 filter paper. These filter papers were air dried and stored individually in Ziplock bags containing silica desiccant beads and refrigerated (-20°C). Parasite detection was screened by ICT malaria *Plasmodium falciparum* cassette test (ICT Diagnostics, South Africa) and positive samples were treated with artesunate-amodiaquine (Camoquin plus).

### G6PD enzyme measurement

All dried blood spots (*n* = 4,144) were detcted for G6PD deficiency by a commercial fluorescence spot test (FST) kit (Guangzhou Micky Medical Instrument Co., China) as previous report [18,19], which was approved by the Chinese Food and Drug Administration (CFDA) (reg. no. CFDA (P) 20112400503). The kits utilized a modification of the classic semi-quantitative Beutler method [20], which test the rate of NADPH generation in mol per min per gm Hb from the chemical reaction catalyzed by G6PD. The cut-off value for this study was set at 2.7U/gHb [19]. The assay was performed according to the manufacturer’s protocol. Then, an aliquot of the lysate of these suspected deficient samples (G6PD value <2.7 U/gHb) was spotted on a Whatman fillter paper, air dried and examined under UV light. Samples from G6PD-defcient subjects showed no or diminished fluorescence as compared with non-deficient samples. G6PD Micky controls (normal and deficient only), provided by Guangzhou Micky Medical Instrument Co., China), were assessed periodically to ensure quality performance of the FST.

### G6PD genotyping

**Fig 2:**
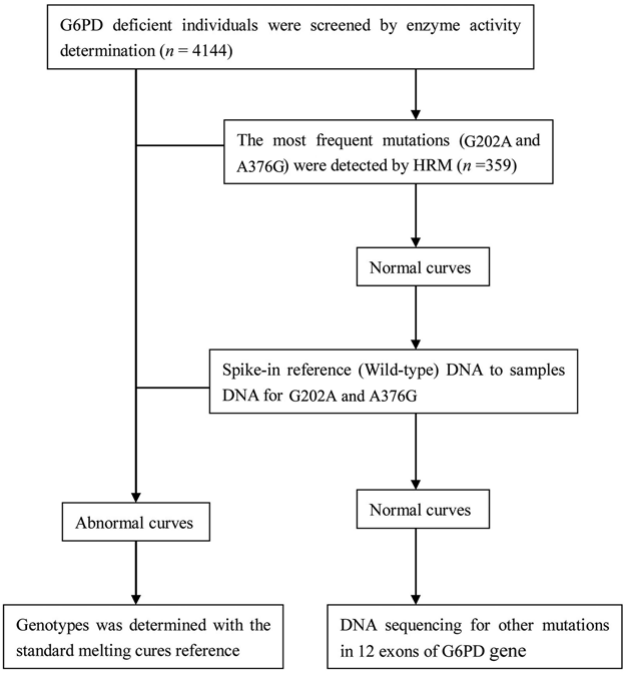
Molecular analysis strategies for screening G6PD mutations.

Genomic DNA was extracted from the G6PD-deficient samples by a DNA blood mini kit (QIAGEN, China). The DNA concentration was measured by UV-2000 spectrophotometry (UNICO, Shanghai), then adjusted to 40 ng/μL for PCR. The molecular analysis strategies for screening G6PD mutations were shown in Fig 2.

All G6PD-deficient samples were analyzed for G202A (c.202 G>A) and A376G (c.376 A>G) mutations by high-resolution melting (HRM) assay as described previously [21]. Primer sequences for HRM genotyping were shown in Table 1. PCR amplification was performed with LightCycle@480 II (Roche Diagnostics) in a total volume of 20 μL, containing 2 μL DNA, 4 μL of 5×PCR buffer, 0.2 μL HotStar Taq, (Takara Biotechnology Dalian Co., China), 1 μL each primer (10 μmol/L), 1.6 μL dNTP (2.5 mM) and 1 μL LC Green plus (Idaho Technology). The cycling conditions for all amplicons were 95°C for 3 min, followed by 35 cycles of 98°C for 10 s, 63°C for 5 s and 72°C for 15 s. Then for melting, fragments underwent denaturalization at 95°C for 1 min, renaturation at 40°C for 1 min, and melting with a continuous fluorescent reading from 60 to 90°C at 25 acquisitions per °C.

**Table 1:** The primers for HRM screening for G6PD A- variant.

Mutations	Exons	Name	Sequence (5’-3’)	Product
G202A	4	P1	TGCCCTCAGGTGGCTGTT	108 bp
		P2	GCTCACTCTGTTTGCGGATGT	
A376G	5	P3	TACCAGCGCCTCAACAGC	99 bp
		P4	GGCAAGGCCAGGTAGAAGA	

Melting curve profiles were generated by increasing the temperature from 65°C to 95°C at 0.05°C/s, and fluorescence was continuously acquired. HRM analysis involved use of LightCycler 480 SW 1.5 (Roche Diagnostics). Normalized melting curves showed the fluorescence signal against temperature, and derivative plots showed melting temperature peaks. The pre-identified DNA including G202A, A376G and wild-type DNA, were used as standard references. When the plots of samples were classified by the standard reference, they were identified as the same genotype of the standard. 12 exons of G6PD gene were amplified and sequenced in G6PD-deficient samples without these two mutations, the primers and conditions were described previously [21].

### Hematological analysis

A total of 1,186 adults were randomly selected from the 4,144 subjects for hematological analysis with the conditions as follows: (1) individuals >16 years old; (2) not pregnant women. Hematological data were collected by an automated blood counter (Systemx F-820; Systemx Corp., Japan). Anemia was defined as hemoglobin level <13.0 g/dL for males and <12.0 g/dL for females. The cut-off values for anemia were >6.0 g/dL, severe; 5.9–9.0 g/dL, moderate; and <9.0 g/dL, mild.

### Molecular diagnosis of hemoglobinopathies

Genomic DNA was extracted by a DNA blood mini kit (QIAGEN, China). DNA concentration was mesured by UV-2000 spectrophotometry (UNICO, Shanghai, China). Then DNA sample concentration was adjusted to 40 ng/μL for PCR.

HbS (c.20 A>T) and HbC (c.19 G>A) were analyzed in the 1,186 subjects by PCR-DNA sequencing. PCR was amplified with Taq polymerase (TaKaRa, Dalian, China) in a MJ Mini Personal Thermal Cycler (Bio-RAD) as described previously [22], with the primers: P1, 5’-AAGGCTGGATTATTCTGAGTC-3’, and P2, 5’-CACTTGCCCGAGTCTGTTT-3’. Reactions involved initial denaturation at 95°C for 3 min, 35 cycles of PCR (95°C for 30 s, 57°C for 30 s and 72°C for 1 min). DNA sequencing was performed by the ABI 3730xL DNA Sequencer (PE Biosystems, CT, USA).

Because most cases of -α^3.7^ thalassemia heterozygotes could not be screened out by the hematological indices (mean corpuscular volume), the 1,186 samples were analyzed for 3 known α-thalassemia deletions (—SEA, -α^3.7^, -α^4.2^) by a commercial thalassemia reverse dot blot (RDB) gene chip (Chaozhou Hybribio Ltd., China) [22,23]. The kit for the gene chip was approved by the CFDA (reg. no. CFDA (P) 20123400399). The assay was performed according to the manufacturer’s protocol.

### Statistical Analysis

Statistical analysis involved use of SPSS 16.0 (SPSS, Chicago, IL). The prevalence of α-thalassemia alleles was calculated from the Hardy-Weinberg formula. Data for the 3 regions on Bioko Island and previous studies were analyzed by chi-square test. *P*<0.05 was considered statistically significant.

## Results

### G6PD deficiency

4,144 subjects, including 2,758 males (66.6%) and 1,386 females (33.4%), received screening for G6PD deficiency. The mean age was 30 year (SD 28.3). *Plasmodium falciparum* malarial parasite was present in 12.6% cases (522/4,144). Amongst these samples, 8.7% (359/4,144) participants were G6PD deficient. The prevalence of males and females were analyzed separately. G6PD deficiency was found in 9.0% (249/2,758) of males and 7.9% (110/1,386) of females (*p* = 0.238). Because of the randomly inactivation of X-chromosome in female, many heterozygous females were expected to have a normal phenotype. These heterozygous females with a normal phenotype could not be detected by FST. Therefore, the detection rate of G6PD deficiency in males was little higher than in females.

**Fig 3:**
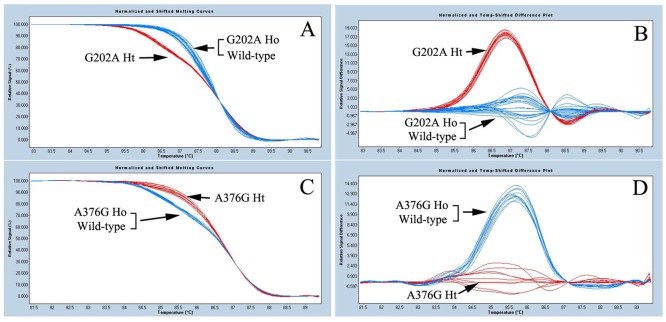
Genotyping of G6PD mutations (G202A and A376G) by high resolution melting (HRM) assay. The arrowhead indicates different genotypes. **A** and **C** are temp-shift melting cures; **B** and **D** are temp-shift difference melting plots. **A** and **B**: G202A mutation; **C** and **D**: A376G mutation.

Of the 359 G6PD-deficient samples, 356 (99.2%, 356/359) were detected with G6PD A^–^ variant (G202A/A376G) by HRM assay. Melting curves for G202A and A376G mutations were shown in Fig 3. PCR-DNA sequencing revealed that the other 3 G6PD-deficient samples were G6PD Betica (T968C/A376G). No any other mutation was found. The number and percentage of different genotypes observed from our samples in both males and females were listed in Table 2.

**Table 2:** The number and percentage of different deficient genotypes from our samples in both males and females.

Genotype	Hemizygote (male)	Heterozygote (female)	Homozygote (female)	Total	Frequency (%)
G6PD A^-^	158	134	64	356	99.2
G6PD Betica	2	1		3	0.8
Total	160	135	64	359	100

### Hemotological data and hemoglobinopathies

Among the 1,186 adults that underwent detection of hemoglobinopathies, the prevalence of anemia was 32.8% (389/1,186): 1.5% severe (*n* = 18), 5.7% moderate (*n* = 67) and 25.6% mild (*n* = 304). The burden of anemia was higher for females than males (37.0%, 183/495 vs 29.8%, 206/691) (*p* = 0.010).

201 HbS heterozygotes, 36 HbC heterozygotes and 2 HbCS double heterozygote were detected from the 1,186 adults. The overall allele frequencies of HbS and HbC were 17.1% (203/1,186) and 3.1% (37/1,186), respectively. Similar to other sub-Saharan African regions, Bioko Island showed a high prevalence of α^+^-thalassemia. The only α-thalassemia mutation observed was α-thalassemia 3.7 kb deletion (-α^3.7^ kb deletion), which accounted for 52.4% (622/1,186) of allele frequencies and was present in a heterozygous state, −α^3.7^/αα (38.1%, 452/1,186) and in a homozygous state, -α^3.7^/-α^3.7^ (7.2%, 85/1,186).

## Discussion

To propose effective public health strategies, reliable epidemiological information for the local population must be obtained. The recent medical communication between Equatorial Guinea and China resulted in an opportunity to address important public health problems, including malaria control and erythrocyte genetic disorders on Bioko Island. This report represented the first detailed population survey assessing the frequencies of classical erythrocyte genetic disorders in this region.

Our survey revealed a high prevalence (8.64%, 359/4,144) of G6PD deficiency on Bioko Island. As compared with other African regions, the prevalence of G6PD deficiency was lower than that of Burkina Faso (31.0%) [24], Congo (22.5%) [25], Mali (15.7%) [26] and Nigeria (21.6%, 23.9% and 24.2%) [27–29] but similar to that report for Ghana (8.5%) [30]. Because G6PD deficiency is notoriously common in malaria endemic areas [1], WHO recommended G6PD testing before giving PQ [31]. However, G6PD testing was rarely done in most regions of Africa including Bioko Island [31]. Similar to many regions of sub-Saharan Africa, G6PD genotype analysis confirmed that almost all deficiency in Bioko Island was caused by G6PD A- variant [6]. The result was similar to those obtained in six African countries [32], and confirms the results shown by previous studies that the G6PD A- was the most common deficient variant in sub-Saharan Africa [33]. This mutation also could be found in the region out of Africa, such as Iraq and Latin America [34, 35], but the prevalence was very lower than in sub-Saharan Africa. The WHO groups the G6PD A- variant into a kind of “mild deficiency” (Class III). Previous studies reported that low dose of PQ (0.25mg/kg) is associated with a red cell survival in the G6PD A^-^ variant five times longer than with the previously recommended 0.75 mg/kg dose [8,36]. Therefore, before the G6PD test was broadly available on Bioko Island, a single dose of 0.25 mg base/kg PQ was recommended to treatment regimens for *P*. *falciparum* malaria in the region.

Sickle cell disorders were frequent on the island, which was consistent with other findings [13, 37]. Modica et al. reported a prevalance of sickle cell disorders on Bioko Island at 20% in 1970 [37]. The crude birth rate was 35.819 (per 1,000 people) on Bioko Island (Data from the Webpage of World Health Organization: Equatorial Guinea Factsheets of Health Statistics 2010) (http://www.afro.who.int/index). The estimated number of pregnancies each year in which the fetus would be at risk for HbS homozygote (HbSS) was 69 (95% confidence interval 52–86). Alpha-talassemia 3.7-kb delection (hetero- and homozygote) was highly prevalent among these participants (52.4%). Indeed, because the Bioko Island Malaria Control Project, started in 2004, had a marked impact on malaria infection and mortality, and malaria was slowly decreasing on the island [38,39], the high prevalence of inherited hemoglobinopathies on the island could become an important disease burden and cause of anemia. However, adequate diagnostic tools in medical services were lacking on Bioko Island. Therefore, the health-system diagnostic capacities needs to be strengthened in African settings to optimize the investigation of genetic hemoglobin disorders and their consequences such as anemia.

In a conclusion, a high frequency of alleles such as G6PD deficiency, HbS, HbC and α-thalassemia associated with malaria resistance were present on Bioko Island. High frequency of G6PD deficiency indicated that the diagnosis and management of G6PD deficiency was necessary on Bioko Island. Before G6PD test was available, a 0.25 mg base/kg PQ single dose was recommended instead of 0.75 mg base/kg PQ dose to treatment regimens for *P*. *falciparum* malaria in this region. An obligatory newborn screening program, prenatal screening and counseling for these genetic disorders, especially HbS, are needed on Bioko Island.
